# Carbendazim trace analysis in different samples by using nanostructured modified carbon paste electrode as voltametric sensor

**DOI:** 10.1371/journal.pone.0279816

**Published:** 2023-01-18

**Authors:** Abbasali Kasaeinasab, Hassan Asilian Mahabadi, Seyed Jamaleddin Shahtaheri, Farnoush Faridbod, Mohammad Reza Ganjali, Fazeleh Mesgari

**Affiliations:** 1 Department of Occupational Health Engineering, School of Medical Sciences, Tarbiat Modares University, Tehran, Iran; 2 Department of Chemistry, University of Louisville, Louisville, Kentucky, United States of America; 3 Department of Occupational Health Engineering, School of Public Health, Institute for Environmental Research, Tehran University of Medical Sciences, Tehran, Iran; 4 Center of Excellence in Electrochemistry, School of Chemistry, University of Tehran, Tehran, Iran; Qatar University, QATAR

## Abstract

Carbendazim (CBZ) as a fungicide is widely used to control fungal diseases in agriculture, veterinary medicine, and forestry. In this study, molecularly imprinted nano-size polymer was synthesized and then combined with multiwalled carbon nanotubes to be used as modifiers for carbon paste electrode to detect carbendazim in water, fruit, agricultural wastewater, and urine samples by using the square-wave technique. Some common ions and pesticides were investigated as interferences in analyte, to study the sensitivity and selectivity of the modified carbon paste electrode for carbendazim. The combination of molecular imprinted polymer and multiwalled carbon nanotubes showed a significant increase in peak current in electrocatalytic activity on electrochemical detection of the carbendazim. The linear range of 1 × 10^−10^ to 5 × 10^−8^ *molL*^−1^ was investigated. The lower detection limit was determined to be 0.2 × 10^−10^ *molL*^−1^, and the relative standard deviation for the target molecule analysis was 2.07%. The result reveals that the modified carbon paste sensor with Multi walled Carbon Nanotubes (MWCNTs) and Molecular Imprinted Polymer (MIPs) can be used easily, without preparation steps that have high selectivity and sensitivity to determine carbendazim in water, fruit, agricultural wastewater, and urine samples.

## 1. Introduction

Pesticides are chemical compounds that are developed and produced to improve food quality, and agricultural productivity and control various diseases and insect pets [1, 2]. Pesticides especially fungicides, are broadly applied in agriculture, the leather industry, industrial antifouling paper-making, fruits storage, urban settings, and a plethora of other uses around the world [3, 4]. Furthermore, pesticides might contaminate the soil, groundwater, and surface from alternate sources and can cause environmental pollution [5]. Carbendazim (CBZ) is a fungicide with solid white crystalline shape and is insoluble in water. Carbendazim (Methyl-2-benzimidazole carbamate, is considered to be a systematic wide-spectrum fungicide and is classified as the benzimidazole family. CBZ has been broadly used in agriculture for pre- and post-harvest, especially in fruits and cereals, to control fungal diseases in plants [6]. CBZ can stay in fruits, crops, vegetables, and the environment for a specific amount of time [7]. Lately, taxological surveys suggested that CBZ as a fungicide can have adverse effects on human health, specifically in reproductive and hormonal areas [8, 9]. By taking them as a low toxicity chemical, the potential effects of fungicides on animal and human health have often been ignored. However, with the development of analytical techniques, data on the toxicity of fungicides in different experimental models has drastically increased [10–12].

The determination of trace amounts of CBZ is a crucial subject, owing to the compound high potential toxicity to animals and human. Thus, having a simple, sensitive, and accurate technique to determine the CBZ in food, environmental, and biological samples have practical significance. Several analytical techniques had been used to detect CBZ, such as spectrophotometry, chemiluminescence, and chromatography [13–18]. Other determination techniques applied for these compounds are enzymatic techniques and immunoassays, and some time these techniques are in combination with mass spectrometry [19, 20]. Furthermore, recently electrochemical techniques have new aspects. These include ease of use, short time analysis, lower costs. and more importantly, high sensitivity. These new aspects used to detect trace level of CBZ have been improved. Due to some difficulties, like sample preparation, expensive instruments, and time-consuming methods the issue of analyzing pesticide residue is a pivotal topic in chemistry, especially in analytical chemistry. Therefore, to determine the pesticide residue, developing simple, low-cost and novel analytical techniques should be considered. These difficulties mostly include the level of skills, and the laboratory equipment necessary for them is substantial. Pesticide analysis is essential in developing countries. On the other hand, instruments such as mass spectrophotometry are very expensive for a wide range of use, also, for this instrument preparation for samples is needed. However, analytical techniques with a short time analysis are required owing to the rising number of samples. Consequently, to determine pesticide residue in different matrixes like food, water, and urine samples, enhanced novel analytical methods in terms of selectivity, precision, and sensitivity have to be taken into account [21, 22].

Modified electrode as a sensor and small instrument, is suitable tools to analyze the residue of pesticides. Due to their low cost, portability, and appropriate size, they are highly proficient devices for on-site analyzing. To improve the sensitivity and selectivity of sensor several types of chemicals like carbon nano materials were applied as modifiers to determine trace levels of CBZ, [23–26]. Besides carbon nanomaterials, Molecular imprinted polymers (MIPs) can be applied as modifier compounds in sensor structure to improve the peak current [22, 27–32]. Synthesis these materials with large molecular adsorption sites create high selectivity and specificity for analyte of interest, which is contributed to thermal, chemical and mechanical stabilities of this polymeric receptor [33, 34]. The features of polymeric receptors rely on several factors, such as the initiator, functional monomer, ratio of monomer to cross-linker, and organic solvent. Therefore, attention must be given to the selecting of these factors to synthesize the best possible polymer with high selectivity [35–38].

However, modified electrode with MIPs and multiwalled carbon nanotubes as a sensor can improve some features, including a prolonged lifetime, and good selectivity, analytical response, sensitivity, and probability. Different electrochemical techniques such as cyclic voltammetry, square wave voltammetry, differential pulse voltammetry, adsorptive stripping voltammetry, differential pulse polarography, and potentiometry can be applied for modified electrodes as electrochemical techniques [39]. It is mentioning that the square wave voltammetry technique has a high sensitivity for detecting chemical analytes [40]. In this study we proposed a highly sensitive nano sensor to determine traces of CBZ in agricultural wastewater, strawberries, and water and urine samples with short analysis time, and ease of use, without having any preparation step.

## 2. Experimental

### 2.1 Instrumentation and chemicals

Methacrylic acid (MAA) and ethylene glycol dimethacrylate (EGDMA) were purchased from Fluka (Buchs, Switzerland). Multiwalled Carbon Nano Tubes (MWCNTs) were purchased from Sigma_Aldrich (Germany). Carbendazim, Atrazine, Oxamyl, Benimyl and 2, 2-azobisisobutyronitrile (AIBN) were purchased from Sigma_Aldrich (Munich, Germany). Paraffin oil, graphite powder (1–2 µm), and all other chemicals such as acetate buffer were purchased from Merck (Germany) and used as received. FT-IR (Perkin Elmer) spectroscopy was used as a spectroscopic framework. Electrochemical measurements were conducted using a Drop Sense electrochemical system (DRP-STAT400-ST40010034) in a 3-electrode configuration, using a carbon paste working electrode, a graphite rod as the counter electrode, and an Ag/AgCl reference electrode (Metrohm, Switzerland). The microstructure of MIP was characterized by scanning electron microscopy (SEM) using a JSM-6380LV instrument (JeolÔ, Tokyo, Japan, 30kV).

### 2.2 Reproduce synthesis procedure

The molecularly imprinted polymer (MIP) was synthesized to be selective for carbendazim. To synthesis MIPs, polymerization was conducted using a precipitation technique to make them nano-size. For the synthesis, carbendazim (0.5 mmol. mL^-1^) was dissolved in 30 mL of chloroform, and then MAA monomer (2 mmolmL^-1^), EGDMA (10 mmolmL^-1^), and AIBN (40 mg) were added as cross-linkers and initiators, respectively. The solution was purged with nitrogen for 10 minutes in sealed atmosphere. Then, polymerization occurred in a water bath at 60°C for 18 hours. Finally, the polymers were dried under vacuum at 60°C. CBZ (as the template) was then removed from the polymer by Soxhlet extraction in methanol for 72 hours [41]. The non-imprinted polymer (NIP) was prepared in an identical manner to the MIP, except that the CBZ template was absent in the polymerization process.

### 2.3 Construction of the sensors

The bare carbon paste electrode (CPE) was prepared by hand, blending graphite powder and paraffin oil at a proportion of 70:30 (w/w %). The blend was homogenized in a mortar for 20 minutes. The resulting paste was stuffed into a plastic cylinder (id: 2 mm, paste deep: 3 cm) in which electrical contact was achieved by a copper wire. The total weight of the paste was 0.1 g. In this study, the multiwalled carbon nanotubes (MWCNTs) and MIPs were used as modifiers. Since the type and ratio of applied components are important to have in the optimum construction of the sensor, we added diverse quantities of modifiers to the paste, and they were packed into the sensor tube. The modified sensor was prepared similarly by blending 42% graphite, 30% paraffin oil, 20% MIP, and 3% MWCNTs (Sensor No. 5 in Table 1). As it is found in Table 1, the various proportions of graphite, paraffin oil, MWCNTs and MIP (or NIP) were used to prepare each of the twelve sensors. Next, the sensor surface was polished using a paper to rub out a thin layer of excess material, so that it be reused after each experiment. Many sensors were prepared with different relative amounts of components to optimize the sensor response.

**Table 1:** The optimization of sensor composition to determine CBZ (1 × 10^−7^ *molL*^−1^).

Sensor No.	Composition of Carbon Paste (Wt. %)					Current (µA)
	Graphite powder	Paraffin oil	NIP	MIP	MWCNT_S_	
**1**	70	30	0	0	0	15.23±0.01
**2**	67	30	0	0	3	25.05± 0.13
**3**	57	30	0	10	3	35.66±0.75
**4**	52	30	0	15	3	44.78± 1.54
**5**	47	30	0	20	3	**49.25± 1.37**
**6**	42	30	0	25	3	46.56± 0.76
**7**	37	30	0	30	3	45.09± 0.95
**8**	57	30	10	0	3	19.06± 1.38
**9**	52	30	15	0	3	17.50± 1.66
**10**	47	30	20	0	3	14.30± 1.09
**11**	42	30	25	0	3	12.10± 1.85
**12**	50	30	0	20	0	29.43± 1.78

### 2.4 Electrochemical procedures

The stock solution of 1 × 10^−3^ *molL*^−1^ CBZ was prepared in ethanol and used for further dilutions by deionized water to provide working solutions. Each modified working electrode (MWCNTs-MIP-CPE) was soaked in the CBZ solution with a certain concentration and pH was adjusted (pH: 4) to extract the CBZ. The pH factor was optimized in the range of 2 to 10 to select the optimum one. All solutions were stirred for 15 minutes at a stirring rate of 600 rpm. The electrochemical cell containing the supporting electrolyte (phosphate buffer 0.1*molL*^−1^, pH: 4) was used for voltammetry experiments. The highest peak current intensities were obtained in amplitude 1.03 V, frequency 20 Hz, and step potential 0.03 V (Optimized parameters). It is worth mentioning that factors affecting the analyte extraction and instrumental parameters were selected based on optimization to obtain the maximum peak current. In fact, parameters such as: the extraction pH, stirring rate of solution, extraction time, analysis pH, deposition potential, and electrolyte concentration, square wave amplitude, and frequency had been previously optimized in different levels, and then selected parameters were used in this study. For example, the step potential of 0.03 V to the working electrode increasing the current intensity due to the accumulation of analyte into the electrode surface. Then, the measurement was completed in the analyzing step via inserting the electrode into a 3 electrodes electrochemical cell with mentioned characteristics. Square wave voltammograms were recorded in the scan range of 0.0 to 0.5 V.

## 3. Results and discussion

### 3.1 Electrochemical characterization of carbendazim

**Fig 1:**
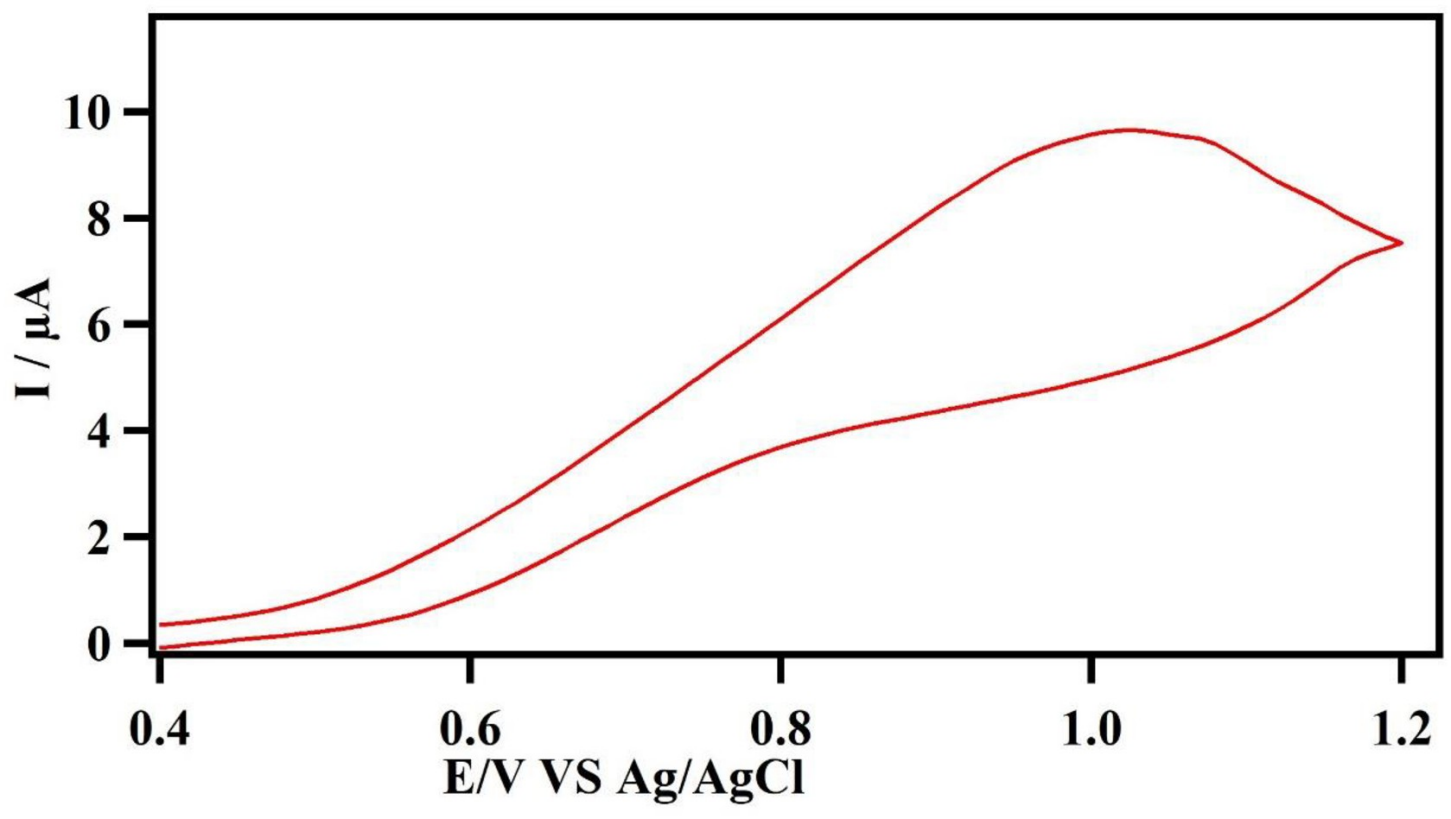
Cyclic voltammetry behavior of CBZ at the surface of bare carbon paste electrode in 0.1 mol*L*^−1^ phosphate buffer electrolyte (scan rate: 100 mV/s).

We investigated the electrochemical behavior of the CBZ with various sensors in different medias First, cyclic voltammograms were obtained on a bare carbon paste electrode without MIPs or NIPs to choose the optimal potential scanning range for CBZ. To reveal the appropriate scan range, the potential was swept from 0 to +1.5. One oxidation peak around 1.03 V was observed and there are no reduction peaks, which shows the chemical irreversibility of the electrochemical process. Fig 1 demonstrates the cyclic voltammogram of CBZ.

### 3.2 Morphological and structural characterization of CBZ

**Fig 2:**
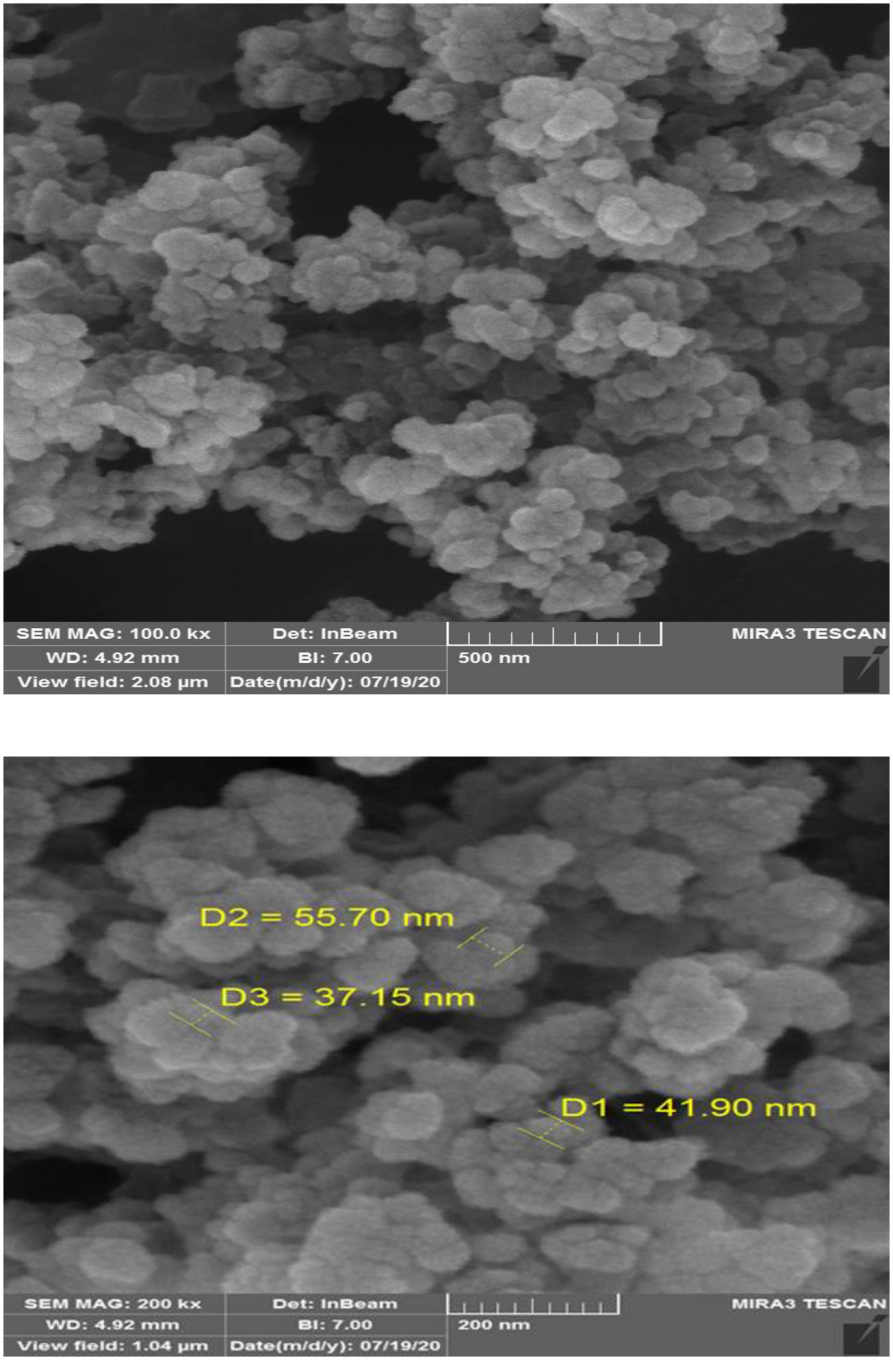
SEM image of molecularly imprinted polymers of the CBZ.

To characterize the fabricated sensor, the characterization of MIPs for CBZ were investigated by SEM and FT-IR. Fig 2 shows the scanning electron microscope (SEM) image of the prepared molecularly imprinted polymers for CBZ. The scanning electron microscopy was done for further investigation of the MIP morphology. According to the observations, the formation of CBZ imprinted particles has been successfully made in nano size and there is a spherical shape with a narrow size distribution.

**Fig 3:**
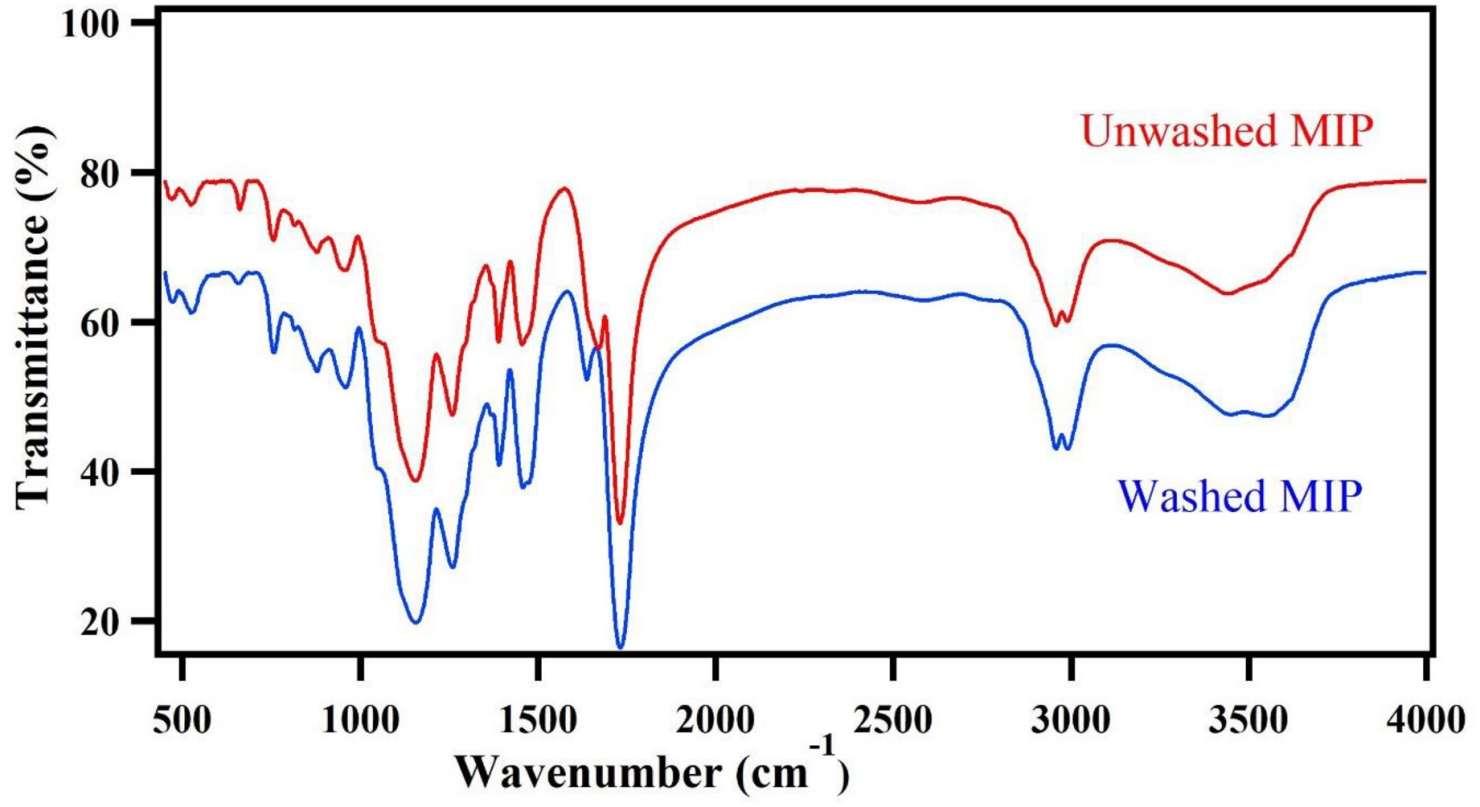
FT-IR spectra of MIP for CBZ, washed MIP, and unwashed.

Furthermore, FT-IR spectroscopic analysis of un-leached and leached CBZ imprinted polymer materials (Fig 3) demonstrates the similarity in the molecular structure of these polymers. As it can be seen, the carboxylic acid OH Stretch vibration in the washed and unwashed MIPs are 2982 and 2991*cm*^−1^, respectively. This displacement toward lower frequencies can be attributed to the interaction of hydrogen with the CBZ in the unwashed MIP. The carbonyl group C = O stretching peak was observed in 1736 and 1741 *cm*^−1^, for washed and unwashed polymers, respectively and it might be related to MAA and EGDMA. The displacement of these peaks and the other one in both polymers supports the idea of interaction between the functional monomer and template molecule. The peaks beyond 1500 *cm*^−1^, placed in the fingerprint region, are complicated to interpret due to many bands overlapping each other.

### 3.3 Effective area of the electrode

The Randles -Sevick equation was applied to evaluate the carbon paste electrode. Cyclic voltammetry with different scan rates was used in a three electrodes electrochemical cell with KCl (0.1M) as a supportive electrolyte and K_3_[Fe (CN)_6_] as a standard sample solution (0.001M) [42, 43]. The transformed detecting surface area was determined to be 0.091 cm^2^, and for bare electrode was 0.045 cm^2^, using the Randles- Sevick equation. The diffusion coefficient (D_0_) from the linear graph of I_P_ VS *v*^1/2^ was found to be 7.2 × 10^−6^ cm^2^ S^-1^.

$$I_{p}=(2.69\times 10^{5})n^{3/2}A{D_{0}}^{1/2}\nu^{1/2}{C_{0}}^{*}$$ (1)

Where *I*_*P*_ is peak current (µA), n is the number of electrons, A is sensing area cm^2^, *D*_0_ is diffusion coefficient (*cm*^2^/*s*), ν is scan rate mv/s *C*_0_ is concentration (*mol/cm*^3^).

It was concluded that MWCNTs-MIPs had the booting effect on the specific surface area and electron transfer compared to the bare electrode.

### 3.4 Electrochemical performance of proposed sensor

**Fig 4:**
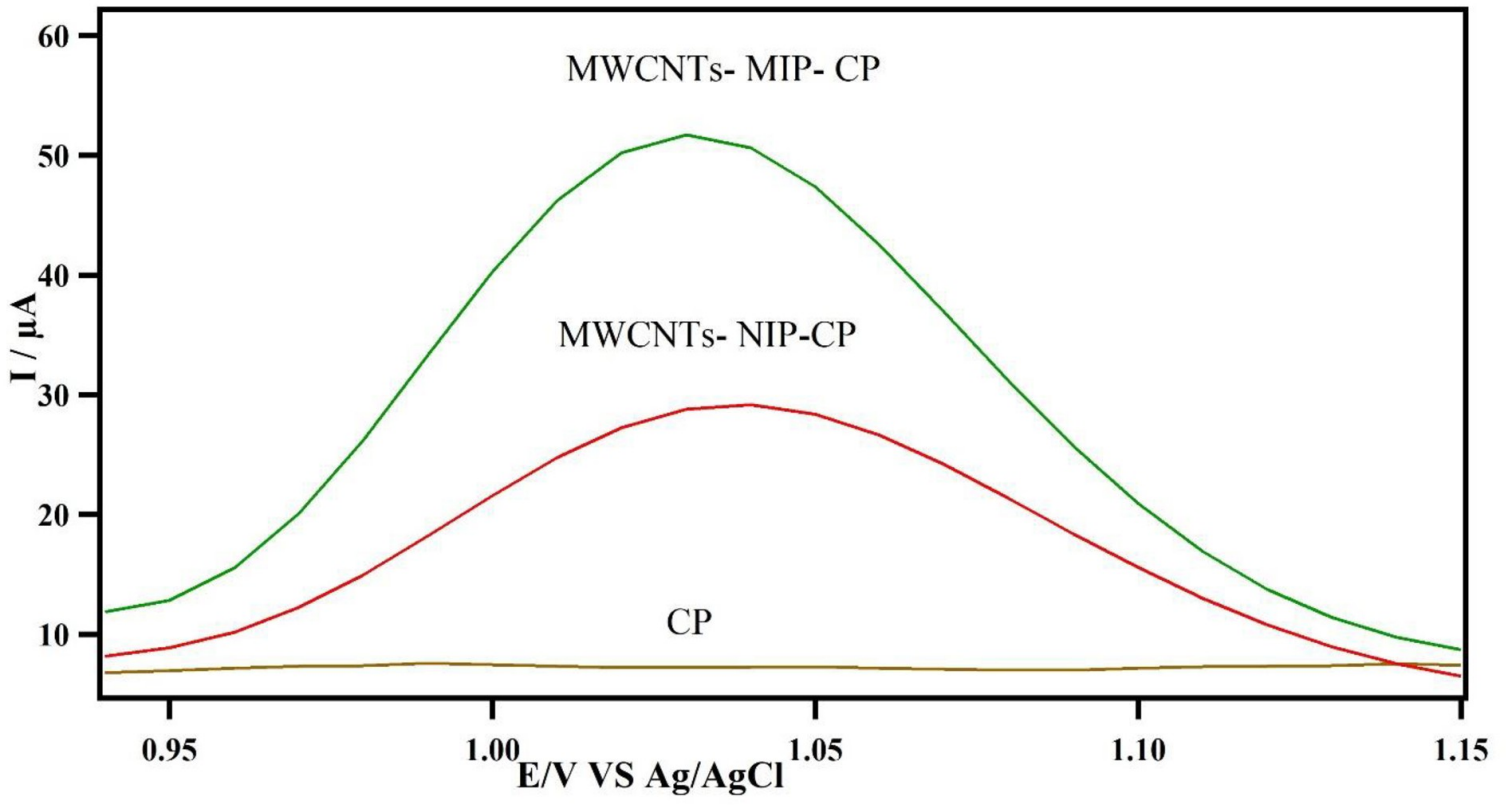
The voltammograms of MWCNTs-MIP-CPE, MWCNTs-NIP-CPE, and CP for CBZ sensor in CBZ 1.0×10^−7^ mol mol*L*^−1^. (Supporting electrolyte: phosphate buffer 0.1mol mol*L*^−1^) (Scan rate: 100 mV/s).

As Fig 4 shows, the voltammetry response of the MWCNTs -MIP-CP and MWCNTs -NIP-CP sensor in terms of their sensitivities were different in terms of CBZ detection in the same concentrations. The result indicated that the sensor with the combination of MWCNTs and MIP nanoparticles (No.5) had a greater current response compared to MWCNTs-NIP nanoparticle sensor. There was a significant difference between the three sensors (MWCNTs -MIP-CP, MWCNTs -NIP-CP, and CP) in respect of affinity to CBZ (P< 0.05). The result showed that the peak current referred to the MWCNTs -MIP-CP sensor is approximately more than twofold and fivefold as much as MWCNTs -NIP-CP and CP respectively, which can be contributed to adding modifiers. Furthermore, the voltagram shows us that the peak current (I_p_) of the CBZ has intensified due to the MIPs and multi-wall carbon nanotubes’ special properties such as their high surface area, dynamic electron transmission capacity. According to these experimental findings, the modified sensor showed effective electrocatalytic properties responsible for strengthening the detection and determination of CBZ in environmental samples. Therefore, for further investigation, this developed sensor was used.

### 3.5 Interference effect and reproducibility

**Fig 5:**
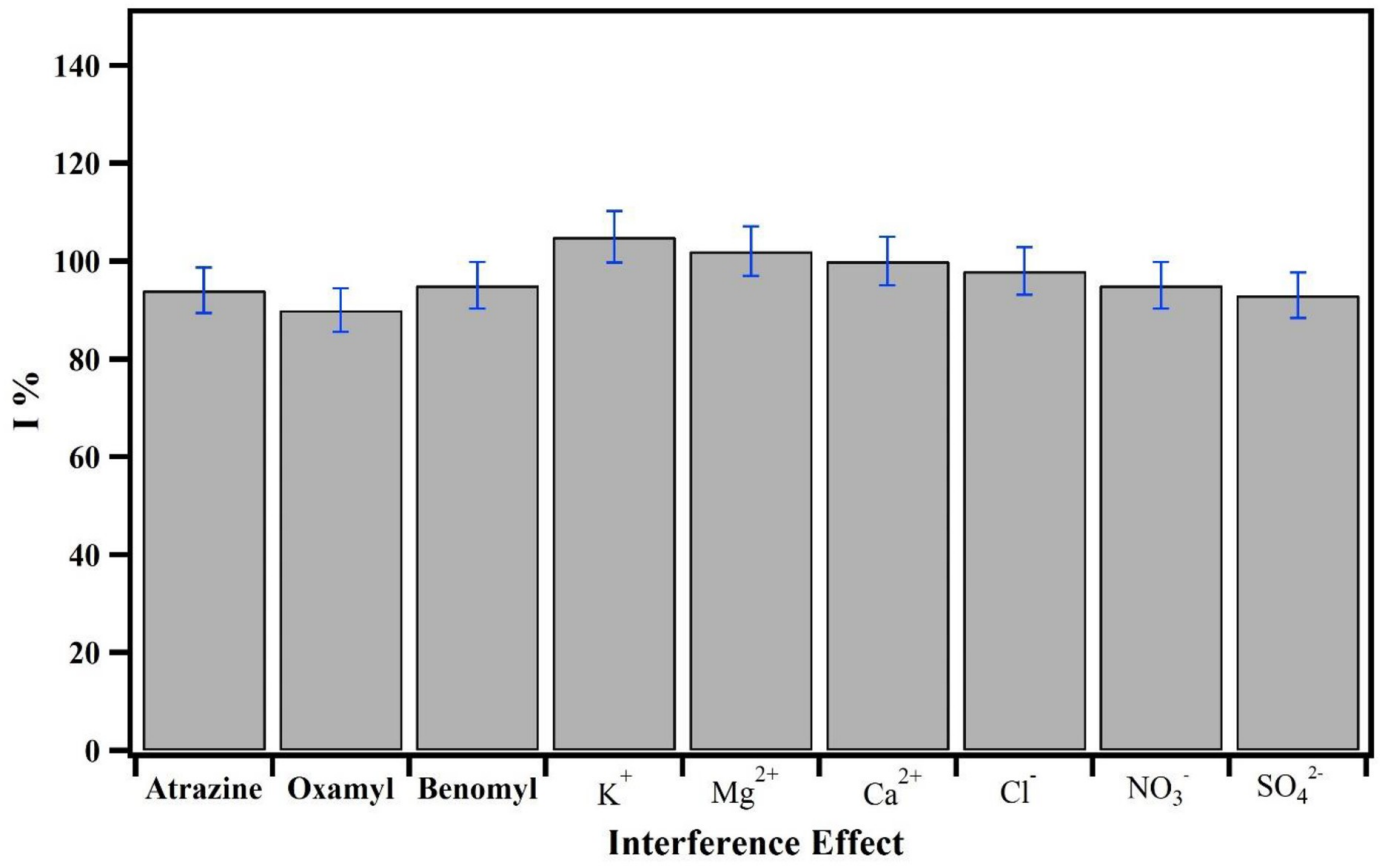
Effects of inorganic ions and several interfering pesticides on detection of CBZ (ΔI %).

Anti-interference capability is a significant factor for an electrochemical sensor. In this study, several possible interfering inorganic ions and several key pesticides were used to evaluate the anti-interference capability of the MWNTs/MIP/CPE sensor to detect CBZ [44]. For this experiment, the interfering inorganic ions in particular cations (*Mg*^2+^ *K*^+^, and *Ca*^2+^) and aqueous anions (*Cl*^−^, *No*_3_^−^, *and S0*_4_^−2^) were added to the certain solution of CBZ, and the concentrations of these inorganic ions were 100 times higher than CBZ. As shown in Fig 5, there were no obvious interferences in the peak current intensity of CBZ after adding these inorganic ions. Also, several pesticides including Atrazine, Oxamyl and Benomyl as interfering agents were added to the CBZ solution. The concentrations of these pesticides were two times higher than those for CBZ. It was clear that no obvious interferences were observed in the simultaneous detection of CBZ after adding interfering pesticides, and the changes of peak current intensity were all below 3.82%. To investigate the reproducibility of the sensor, five consecutive measurements were recorded by to determine the current response in PBS buffer containing 0.1 *µmolL*^−1^ CBZ, and the relative standard deviation (RSD) was <2.7%. This RSD indicated that the MWNTs/MIP/CPE sensor demonstrated acceptable reproducibility in the simultaneous detection of CBZ. This indicates that the metal ions and other pesticides had no impact on the CBZ peak current along with the selective determination of CBZ in the presence of other pesticide molecules and metal ions at the altered electrodes. These results supported the assertion that this sensor offers strong anti-interference effects and suitable reproducibility which could be used for simultaneous detection of CBZ in real samples. Favorable repeatability and reproducibility of the electrode are the main factors in using the modified electrode to a larger scale for applications in the future. Therefore, the reproducibility of the modified electrode was examined in the alternate interval of a day by recording the electrochemical response of CBZ. It has been discovered that the MWNTs-MIP was capable of emitting signals with good reproducibility that had an RSD of 0.91% (N = 4). The stability of the CBZ-MWNTs-MIP had been examined for electrochemical determination to evaluate the capability and repeatability. The developed electrode was in a sealed container for ten days. The findings revealed 95.0–97.0% recovery value which indicated the efficacy of the sensor.

### 3.6 Detection of carbendazim by square wave voltammetry (SWV)

**Fig 6:**
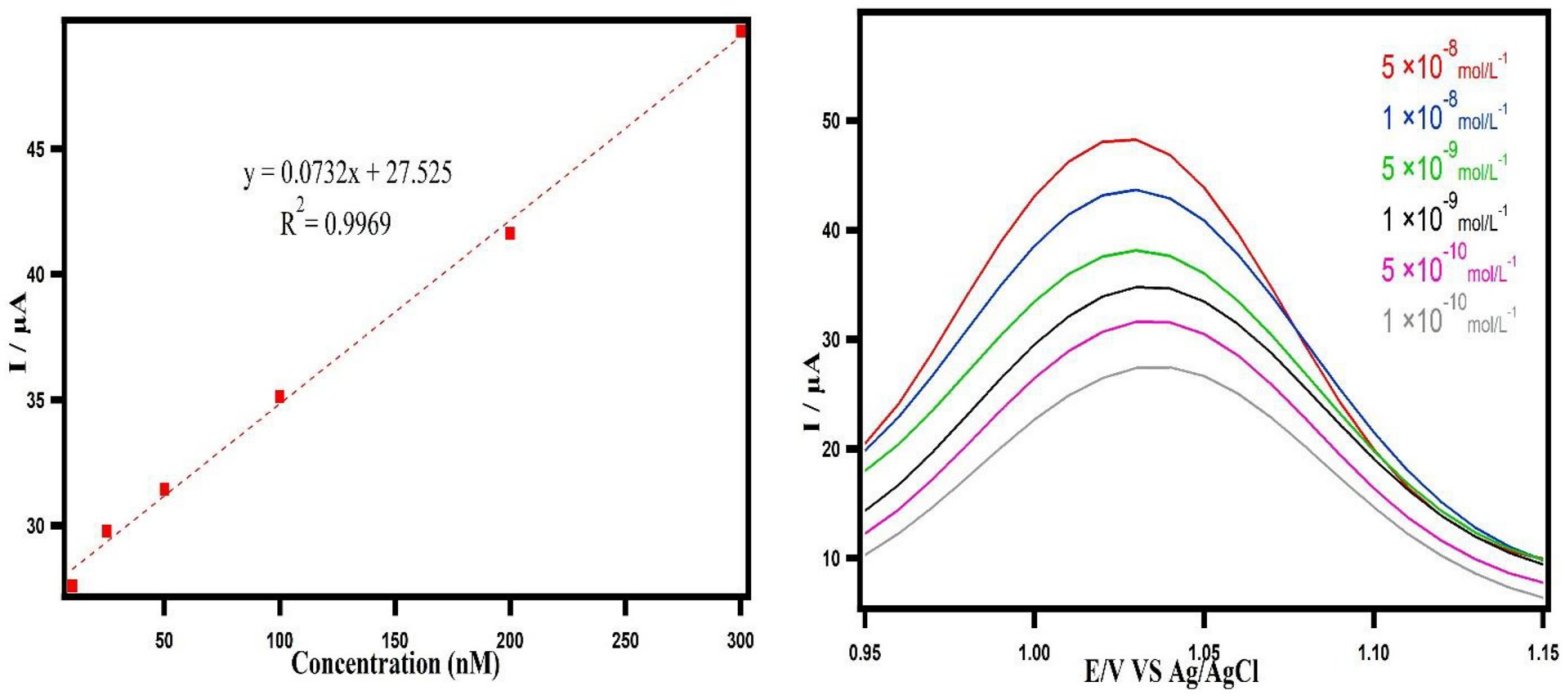
Calibration curve plotted for voltametric determination of CBZ by using MWNTs/MIP/CP sensor.

The integral electrochemical investigation gives us vital information on the electrode processes. The SWV technique was used to identify CBZ. The SWV is one of the fastest and most sensitive pulse voltammetry techniques. The detection limits can be compared with those of chromatographic and spectroscopic techniques [45]. Thus, the concentration of CBZ was determined by using the SWV technique. The noted modified electrode demonstrated a great electrochemical enhancement of CBZ. The increasing current intensity is attributed to the large surface area of the electrode; therefore, it exhibits the effective fabrication of the MWNTs-MIP’s impressive properties, as well as large specific surface area, faster electron transferring capacity, and there is an increase in the oxidation current of the CBZ at the MWNTs-MIP. Fig 5 indicates detection of CBZ with MWNTs/MIP/CP sensor which was carried out by square wave voltammetry in 0.1 *molL*^−1^ phosphate buffer pH 4.0. The analytical curve (Fig 6) was linear for the concentration range of 1 × 10^−10^ to 5 × 10^−8^ *molL*^−1^. The linear equation was *y* = 0.0732*x* + 27.525 with a correlation coefficient of 0.9969. Furthermore, lower limit of detection (LOD) was found to be 0.2 × 10^−10^ *molL*^−1^ (S/N = 3), which is lower than the previous works to the best of our knowledge. Table 2 exhibits the relative study of detection limit of CBZ with formerly reported research. The LOD was calculated by the Miller and Miller statistical method [46].

**Table 2:** Limit of detection of the present and some of the previously published studies.

Modified electrodes	Linear range (*mol M*^−1^)	LOD (mol L^-1^)	Ref.
**NPG/GCE** ^1^	5 × 10^−6^ − 1.5 × 10^−4^	2 × 10^−7^	[44]
**SiO_2_/ MWCNTs/ GCE** ^2^	2 × 10^−7^ − 4 × 10^−6^	5.6 × 10^−8^	[47]
**NP-Cu/RGO/GCE** ^3^	2 × 10^−7^ − 4 × 10^−6^	9 × 10^−8^	[48]
**TCP/CPE** ^4^	5 × 10^−8^ − 1 × 10^−4^	3 × 10^−8^	[49]
**ZnFe_2_O_4_/ SWCNTs/GCE** ^5^	5 × 10^−7^ − 1 × 10^−4^	9 × 10^−8^	[50]
**CPE/15FS@Ag** ^6^	8 × 10^−8^ − 3 × 10^−6^	9.4 × 10^−10^	[13]
**La-Nd_2_O_3_/CPE** ^7^	8 × 10^−8^ − 5 × 10^−5^	2.7 × 10^−8^	[51]
**MWCNTs/Ca-ZnO-CPE**	1 × 10^−8^ − 4.5 × 10^−7^	4.68 × 10^−9^	[52]
**CPE-RGO/Biochar**	3 × 10^−8^ − 9 × 10^−7^	2.3 × 10^−9^	[53]
**MWCNTs/MIP/CPE**	1 × 10^−10^ − 5 × 10^−8^	0.2 × 10^−10^	Present Study

^1^. Nanoporous gold-GCE.

^2^. Silicon dioxide-multi walled carbon nanotubes-GCE.

^3^. Nanoporous copper-reduced graphene oxide-GCE.

^4^. Tricresyl phosphate-CPE.

^5^. Spinel ferrite-single walled carbon nanotubes-GCE.

^6^. Silver nanoparticles on fumed silica-CPE.

^7^. La-doped Nd2O3-CPE.

### 3.7. Determination of CBZ in real samples

The efficiency and accuracy of the modified electrode are identified by analyzing the spiked sample using the SWV technique. The analyses conducted for agricultural wastewater, strawberries, tap water, and urine samples were selected as real samples to evaluate the applicability and practical feasibility of the method. Real samples were treated with the same procedure by spiking a certain amount of CBZ standards. The modified electrode was sucked into the sample solutions. Then the electrode was sucked up in the buffer solution for further analysis through the voltammetric procedure. The concentration of CBZ in each sample was determined. Finally, CBZ recoveries were performed. Table 3 shows the results for the recovery of CBZ in spiked samples. The value nearing 100.00% of recovery tests validated the superb accuracy of the modified electrochemical sensor, showing the efficient determination of CBZ in authentic samples free of any interference effect. As it can be seen, the proposed electrode along with voltammetry procedure is sufficiently acceptable for environmental and biological monitoring of CBZ. It is worth mentioning that this procedure is simple, and it gives limits of determination in trace levels. High sensitivity, good reproducibility, and simple instrumentation are some other advantages of this modified electrode. So, this method could be easily applied for the determination of carbendazim in real samples. As we observe in Table 3, the changed electrode had a satisfactory recovery for agricultural wastewater, urine, strawberries, and tap water. This excellent relative recovery is attributed to the large surface area and fast electron transfer in the modified electrode.

**Table 3:** Recovery studies of CBZ in spiked real samples (n = 3).

Sample	Proposed sensor				HPLC			
	Added (ng/ml)	Found (ng/ml)	RSD (%) (N = 3)	Relative Recovery (%)	Added (ng/ml)	Found (ng/ml)	RSD (%) (N = 3)	Relative Recovery (%)
**Agriculture Wastewater**	0	ND*	0	0	0	ND	0	0
	5	4.92	2.41	98.40	5	4.96	1.89	99.2
	50	49.67	3.24	99.35	50	52.05	2.12	104.1
	500	496.35	1.80	99.27	500	512.5	1.52	102.5
	1000	995.10	2.89	99.51	1000	1033	3.17	103.3
**Strawberry**	0	ND	0	0	0	ND	0	0
	5	4.98	1.89	99.61	5	5.17	3.11	103.4
	50	49.85	3.32	99.70	50	49.3	2.54	98.6
	500	497.85	2.53	99.57	500	506	2.31	101.2
	1000	997.90	2.45	99.79	1000	971	2.01	97.1
**Tap Water**	0	ND	0	0	0	ND	0	0
	5	4.97	2.37	99.50	5	5.06	1.93	101.2
	50	49.98	3.20	99.96	50	51.2	261	102.5
	500	495.75	2.81	99.15	500	521.5	2.38	104.3
	1000	994.70	3.12	99.47	1000	1001	3.22	100.1
**Urine**	0	ND	0	0	0	ND	0	0
	5	4.78	2.40	95.70	5	4.98	2.83	99.7
	50	47.91	2.36	95.83	50	51.6	3.12	103.2
	500	484.55	3.27	96.91	500	496	2.18	99.2
	1000	967.91	1.39	96.79	1000	1021	3.05	102.1

*ND: No Detection

## 4. Conclusions

In this work, the molecularly imprinted polymer and multi-walled carbon nanotubes were used as modifiers in the development of a highly selective sensor to be used for the determination of CBZ. It was shown that the MIP had a substantial effect on the sensor selectivity. We introduced a suit sensor for detection of CBZ because of the special characteristics of MWNCT_S_ and MIP (nano)-CP electrode. Through the examination, the selectivity of the MIP (nano)-CP electrode for the molecule of interest was improved according to the higher surface areas of the MIP (nano)-CP electrode rather than the CP electrode without modification. The linear range of 1.0×10^−10^ to 5.0×10^−8^ *molL*^−1^ is acquired based on the optimized test circumstances, and the limit of detection was obtained 0.2×10−^10^ mol.L^-1^. The developed sensor was successfully applied for the detection of CBZ in different authentic samples with no significant matrix interference. The MWCNTs was in combination with MIP running as a sensitive, selective sensor by preconcentration in the carbon paste followed by electrochemical analysis. This MIP (nano)-CP modified electrode has a great day-to-day reproducibility, stability for at least one month, and a low nM detection limit. Further examination will be aimed at enhancing the selectivity, and testing MIPs for other types of pesticides and toxic chemicals of interest in the environment for its reliability and excellent accuracy. The novel MIP (nano)-CP electrode shows a new approach to this sensor with great sensitivity and selectivity and has indicated the need for similar surveys for electrochemical sensors.
